# Identification and Determination of *Aconitum* Alkaloids in *Aconitum* Herbs and *Xiaohuoluo Pill* Using UPLC-ESI-MS

**DOI:** 10.3390/molecules170910242

**Published:** 2012-08-27

**Authors:** Ping Cui, Han Han, Rui Wang, Li Yang

**Affiliations:** The Ministry of Education (MOE) Key Laboratory for Standardization of Chinese Medicines, Shanghai University of Traditional Chinese Medicine, Shanghai 201210, China; Email: cuiping426@126.com (P.C.); pashanhan@126.com (H.H.); yangli7951@hotmail.com (L.Y.)

**Keywords:** UPLC-ESI-MS, *Xiaohuoluo pill*, *Aconitum* alkaloids, multi-component quantitative, quality control

## Abstract

A rapid, specific, and sensitive ultra-performance liquid chromatography-electrospray ionization-mass spectrometry (UPLC-ESI-MS) method to examine the chemical differences between *Aconitum* herbs and processed products has been developed and validated. Combined with chemometrics analysis of principal component analysis (PCA) and orthogonal projection to latent structural discriminate analysis, diester-diterpenoid and monoester-type alkaloids, especially the five alkaloids which contributed to the chemical distinction between *Aconitum* herbs and processed products, namely mesaconitine (MA), aconitine (AC), hypaconitine (HA), benzoylmesaconitine (BMA), and benzoylhypaconitine (BHA), were picked out. Further, the five alkaloids and benzoylaconitine (BAC) have been simultaneously determined in the *Xiaohuoluo pill*. Chromatographic separations were achieved on a C_18_ column and peaks were detected by mass spectrometry in positive ion mode and selected ion recording (SIR) mode. In quantitative analysis, the six alkaloids showed good regression, (*r*) > 0.9984, within the test ranges. The lower limit quantifications (LLOQs) for MA, AC, HA, BMA, BAC, and BHA were 1.41, 1.20, 1.92, 4.28, 1.99 and 2.02 ng·mL^−1^, respectively. Recoveries ranged from 99.7% to 101.7%. The validated method was applied successfully in the analysis of the six alkaloids from different samples, in which significant variations were revealed. Results indicated that the developed assay can be used as an appropriate quality control assay for *Xiaohuoluo pill* and other herbal preparations containing *Aconitum* roots.

## 1. Introduction

The genus *Aconitum*, under which there are many plant species, is one of the most important genera of medicinal plants in China. *Radix Aconiti* (RA), the dried maternal root of *Aconitum carmichaeli* Debx., and *Radix Aconiti Kusnezoffii* (RAK), the dried earthnut root of *Aconitum kusnezoffii* Reichb. RA and RAK, being the most significant representatives of *Aconitum* [1,2], have been used widely in many Chinese medicinal preparations for eliminating wind and dampness, and for warming the channels to relieve pain. The main constituents of *Aconitum* spp. are C_18_-, C_19_-, C_20_-diterpenoid-type and other types of alkaloids. The highest content is of the C_19_-diterpenoid-type alkaloids [3,4], which is composed mainly of the *Aconitum* alkaloids, which comprise the following three main types of alkaloids: diester-diterpenoid alkaloids, such as aconitine (AC), mesaconitine (MA), and hypaconitine (HA); monoester alkaloids, such as benzoylaconine (BAC), benzoylmesaconine (BMA), and benoylhypaconine (BHA); and amine alcohol-type alkaloids [5,6,7]. *Aconitum* alkaloids have anti-inflammatory [8,9], anti-cancer [10,11], anti-ischemic and anoxic, anti-pyretic and analgesic, and immunoregulation [12,13,14] functions. In recent years, because of the large number of *Aconitum* species on Chinese market, mainly *Aconitum carmichaeli* Rchb., *A.vilmorinianum* Kom., *A.karakolicum* Rapcs., *A.hemtkyanum* Pritz., *A.paniculigerum* Nakai and *A. austoryunnanense* W.T. Wang, which are confusingly used as *Aconitum*, the very common adulteration and substitution of the original species cannot be indentified or distinguished by the conventional methods of identification and microscopic identification of powders [1,15,16]. Moreover, due to the significant biological activity and the toxicity of its alkaloids, *Aconitum* has attracted more and more the attention of researchers [17,18].

The high toxicity levels of *Aconitum* are considered to be derived from its diester-diterpenoid alkaloids. Having a spicy taste, the raw roots of *Aconitum* cannot be ingested directly. The herbs must be processed properly (by hydrolysis) to decrease their toxicity. Preparations of certain *Aconitum* species native to Asia are indispensable materials in Traditional Chinese Medicine (TCM). The processing of *Aconitum* is done by soaking or heating it in alkaline or water solution [19,20,21]. The *Chinese Pharmacopoeia (2010 Edition)* and other literature have shown that during processing, hydrolysis of ester groups decreases toxicities, as highly toxic diester-diterpene alkaloids are hydrolyzed to less toxic monoester or amine alcohol-type alkaloids. This process has no significant impacts on bioactivity and pharmacological effects [1,22,23].

*Xiaohuoluo pill*, derived from TCM and composed mainly of *Radix Aconiti Preparata* (RAP) and *Radix Aconiti Kusnezoffii Preparata* (RAKP) as main drugs, accounting for 42% of the entire prescription, has been used clinically in China for the treatment of wind cold damp impediment, limb pains, and numbness [1,24,25,26]. In the past several decades, studies have indicated that the chemical constituents of *Xiaohuoluo pill* are abundant and complex to analyze, that the main bioactive ingredients are monoester-type alkaloids and that its main toxic ingredients are diester-diterpenoid alkaloids [27,28]. Therefore, the development of a rapid, valid, and sensitive method to simultaneously, qualitatively, and quantitatively assess the *Aconitum* alkaloids in *Xiaohuoluo pill* is necessary and significant to ensure its safety and effectiveness in the areas of clinical drug use and quality control.

Many methods, such as high-performance liquid chromatography (HPLC) [29,30,31,32], thin-layer chromatography (TLC) [33,34], gas chromatography-tandem mass spectrometry (GC-MS), and liquid chromatography-tandem mass spectrometry (LC-MS) [35,36,37,38], have been established for the qualitative and quantitative inclusion of *Aconitum* alkaloids. Qiao *et al.* assessed the consistency and differences in the products of Caowu by a rapid resolution liquid chromatographic (RRLC) fingerprint, whereby 11 main aconitum alkaloid peaks were picked out and showed great potential for extensive quality control and safely evaluation of raw and processed Caowu [39]. Moreover, a recently published profiling approach was successfully applied to evaluate chemical constitution between co-decoction and mixed decoction of *Radix aconiti* and *Pinellia praeparata* using ultra performance liquid chromatography coupled with time-of-fight mass spectrometry (UPLC/Q-TOFMS) [40]. Although these methods make it possible to visually differentiate the different chromatograms, however, the process is subjective and does not provide a simultaneous quantitative and qualitative analysis of *Aconitum* herbs and their preparations. Studies that combine chromatographic fingerprinting and multi-ingredient quantification by ultra-performance liquid chromatography-tandem mass spectrometry (UPLC-MS) for the quality control of the *Xiaohuoluo pill* have not been reported.

This study aimed to reveal the *Aconitum* alkaloids in *Aconitum* herbs and their quality control. A simple, accurate, and practical ultra-performance liquid chromatography-electrospray ionization-mass spectrometry (UPLC-ESI-MS) method was developed for the simultaneous identification and determination of *Aconitum* alkaloids. The chemical fingerprints of *Aconitum* and the *Xiaohuoluo pill* were established and investigated using principal component analysis (PCA). The contents of three highly toxic diester-diterpene alkaloids, called AC, MA, and HA, and their hydrolyzates, called BAC, BMA, and BHA, were quantified in *Xiaohuoluo pill*. The combination of chromatographic fingerprint analysis and the simultaneous determination of the six *Aconitum* alkaloids offer a more comprehensive strategy for the quality evaluation of *Aconitum* and *Xiaohuoluo pill*.

## 2. Results and Discussion

### 2.1. LC-MS Fingerprints

#### 2.1.1. UPLC-ESI-MS Fingerprint Analysis of *Aconitum* Herbs and Processed Products

**Figure 1:**
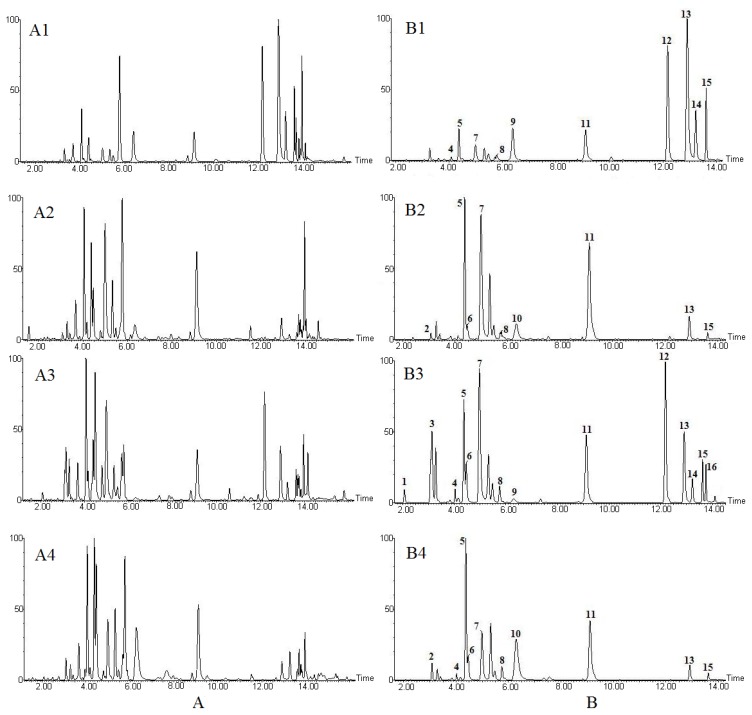
(**A**) UPLC-ESI-MS total ion chromatogram (**B**) and extracted ion chromatogram from *Aconitum* herbs and processed products. (**A1**, **B1**) *Radix Aconiti kusnezoffii*, (**A2**, **B2**) *Radix Aconiti Kusnezoffii Praeparata*, (**A3**, **B3**) *Radix Aconiti*, and (**A4**, **B4**) *Radix Aconiti Praeparata*. Peaks 1 to 16 are shown in Table 1.

The chemical fingerprint of *Aconitum* herbs and processed products was determined and developed using the UPLC-ESI-MS method (Figure 1A). To examine chemical differences between the samples, chromatographic profiling, combined with PCA using the MarkerLynx^TM^ XS software (Waters, Manchester, UK), was exploited. Each variable (data point) in the loading plot represented the loading of the parent ion intensity at that retention time.

**Figure 2:**
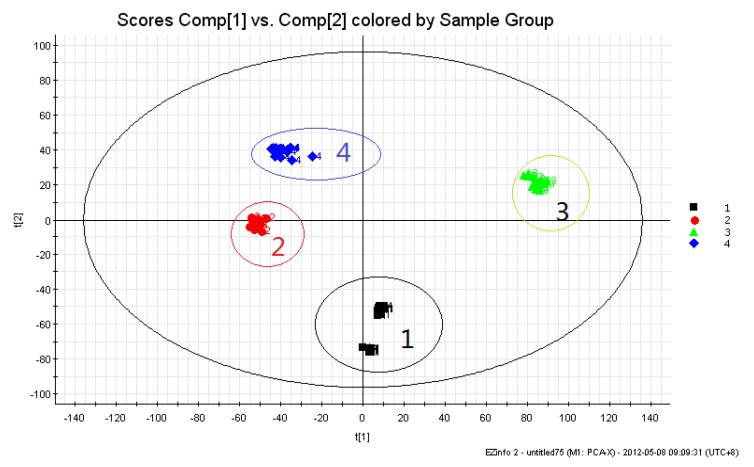
PCA score plot: (**1**) *Radix Aconiti* (RA); (**2**)* Radix Aconiti Praeparata* (RAP); (**3**) *Radix Aconiti Kusnezoffii* (RAK); and (**4**) *Radix Aconiti Kusnezoffii Praeparata* (RAKP).

The PCA result indicated that all samples of *Aconitum* herbs and processed products were clearly clustered into four different groups, where each sample was represented by a marker, so that observations in the same cluster were, in some sense, similar. A score plot derived from PCA is shown in Figure 2. Differences in chemical constituents between RAK and RA might be one of the most important factors in the classification. Besides, crude herbs and processed products were distributed on both sides of the axis, where crude herbs occupied the right side and processed products the left. This result indicated that the chemical constituents of *Aconitum* herbs were varied and different after undergoing the traditional processing.

In the above-mentioned case, a significant separation between the two sets of RAK and RAKP samples was observed in the PCA score plot, which was driven by a number of alkaloids, indicating that the profiling chromatographs of RAK samples were distinct from those of the RAKP samples.

**Table 1:** Sixteen discriminatory chemical markers of *Aconitum* herbs and processed products.

Peak No.	*t*_R_ (min)	*m/* *z*	Molecular Formula	Identification	VIP
1	2.07	408.4	C_23_H_37_NO_6_	cammaconine ^b^	3.91
2	3.08	424.4	C_23_H_37_NO_6_	senbusine A ^b^	5.55
3	3.14	378.4	C_22_H_35_NO_4_	aconosine ^b^	7.62
4	4.04	454.5	C_24_H_39_NO_7_	delcisine ^b^	6.43
5	4.37	408.4	C_23_H_37_NO_6_	isotalatizidine ^b^	7.37
6	4.45	422.5	C_24_H_39_NO_5_	talatizamine ^b^	9.46
7	5.00	590.7	C_31_H_43_NO_10_	benzoylmesaconitine ^a^	9.29
8	5.76	438.5	C_24_H_39_NO_6_	neoline ^b^	8.21
9	6.34	360.4	C_22_H_33_NO_3_	lepenine ^b^	5.68
10	6.38	574.7	C_31_H_43_NO_9_	benzoylhypaconitine ^a^	6.63
11	9.04	358.3	C_22_H_31_NO_3_	songorine ^b^	5.33
12	12.18	632.7	C_33_H_45_NO_11_	mesaconitine ^a^	9.12
13	12.91	616.8	C_33_H_45_NO_10_	hypaconitine ^a^	8.8
14	13.22	646.8	C_34_H_47_NO_11_	aconitine ^a^	5.59
15	13.61	630.8	C_34_H_47_NO_10_	deoxyaconitine ^b^	7.94
16	13.75	402.4	C_24_H_35_NO_4_	lucidusculine ^b^	3.59

^a^ Alkaloids identified by comparing retention times and MS data with those of reference compounds; ^b^ Alkaloids identified by comparing MS data with those reported in literature.

Meanwhile, variable importance in projection (VIP) from MarkerLynx^TM^ XS was screened, with the purpose of extracting the featured beneficial chemical markers for classification. Then, sixteen alkaloids were picked out from the chromatogram at retention times from 2.07 to 13.75 min (Figure 1B). Among them, five compounds were identified unequivocally as benzoylmesaconitine (BMA, 7), benzoylhypaconitine (BHA, 10), mesaconitine (MA, 12), hypaconitine (HA, 13) and aconitine (AC, 14) by comparing retention times and MS data with those of the reference compounds. Other compounds were tentatively identified by comparing MS data with those reported in literature [17,18,38,39,40,41,42,43]. Retention time values, VIP values, and mass data of the deduced compounds from the peaks are summarized in Table 1. Meanwhile, results showed that the important chemical markers of toxicity and bioactivity included three diester-diterpenoid alkaloids (MA, AC, and HA) and two monoester alkaloids (BMA and BHA) in the four groups, which have significantly higher VIP values and play an important role in classification.

#### 2.1.2. Changes in Chemical Components of *Aconitum* Herbs and Processed Products

**Figure 3:**
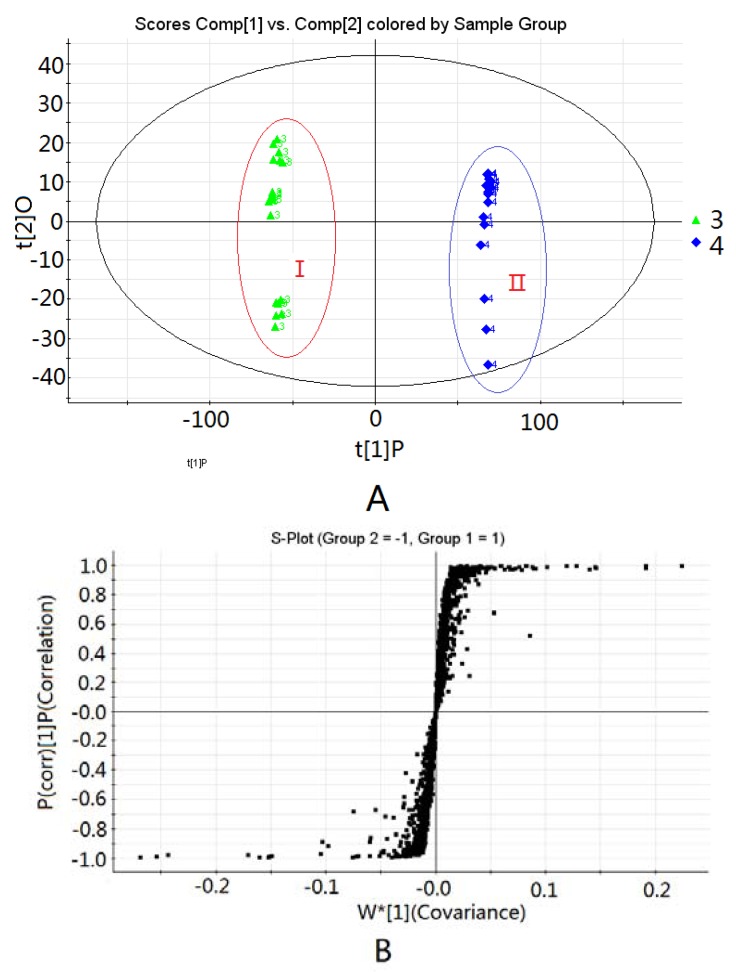
(**A**) OPLS-DA score plot and (**B**) S-plot obtained from the groups of RAK and RAKP (**I**) *Radix Aconiti Kusnezoffii* and (**II**) *Radix Aconiti Kusnezoffii Praeparata*.

To examine chemical differences and pick out potential discriminatory markers between RAK and RAKP, supervised orthogonal projections to latent structures-discriminant analysis (OPLS-DA) statistical model was applied and carried out. In OPLS-DA models, whole variations are separated into orthogonal and correlation variations to better improve the interpretational ability of resulting models and reduce the dimension [44,45]. In Figure 3A, all the samples are separated in the OPLS-DA model and the points of two groups are scattered, to some extent, from the score plot, which is caused mainly by traditional processing.

The S-plot of the OPLS-DA model was used because it helps indentify statistically significant metabolites, and is employed to reveal and identify the discriminatory biomarkers that contribute to sample set separation. Markers, which have both significant covariance and correlation values, are considered as potential markers that discriminate between groups [46]. From the S-plot, variables (markers) with larger VIP values are more relevant in sample classification [47,48,49]. In Figure 3B, alkaloids with larger covariance and correlation values are selected as potential chemical markers to discriminate between the two groups. Meanwhile, discriminatory variables obtained from larger VIP values are helpful in demonstrating the discriminatory variables. Therefore, eight alkaloids with the largest VIP values are picked out. Their average intensities in both RAK and RAKP groups are displayed in Table 2.

**Table 2:** First eightdiscriminatory alkaloid markers in RAK and RAKF.

Peak No.	*t*_R_ (min)	Identification	VIP
13	12.91	hypaconitine	13.70
12	12.18	mesaconitine	12.93
15	13.61	deoxyaconitine	12.43
7	5.00	benzoylmesaconitine	11.41
4	4.04	delcisine	9.75
5	4.37	isotalatizidine	9.75
14	13.22	aconitine	8.71
9	6.34	lepenine	7.74

Examples of discriminatory alkaloids include HA, MA, and AC, with VIP values of 13.70, 12.93, and 8.71, respectively; and BMA, with a VIP value of 11.41. They are significant markers which influence sample discrimination, and could be the chemical markers that show the effect differences between the two groups.

The same procedure was used in the study of RA and RAP. Examples of discriminatory alkaloids include MA and HA, with VIP values of 10.99 and 6.32, respectively, and BHA and BMA, with VIP values of 7.43 and 6.27, respectively. They are significant markers which influence sample discrimination and could be the chemical markers that show the effect differences between RA and RAP.

Through the PCA and OPLS-DA analysis, the content of diester-diterpenoid alkaloids decreased, while the content of monoester-diterpenoid alkaloids increased during processing. Apparently, highly toxic diester-diterpenoid alkaloids have been hydrolyzed to become low toxicity monoester alkaloids, which strongly indicated that traditional drug processing causes a significant change in the chemical components in *Aconitum* herbs and processed products [17,18,19,20].

#### 2.1.3. Chemical Fingerprint of *Xiaohuoluo Pill*

**Figure 4:**
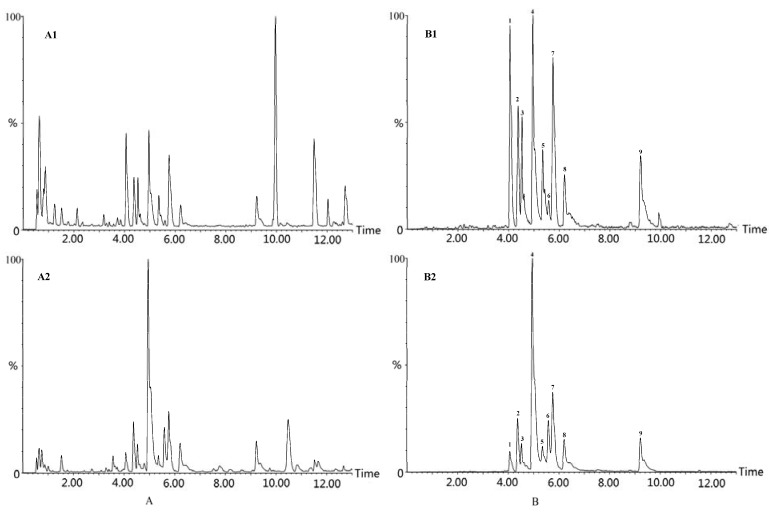
(**A**) UPLC-MS total ion chromatogram and (**B**) extracted ion chromatogram from the *Xiaohuoluo pill*. (**A1**, **B1**) Concentrated pill and (**A2**, **B2**) honey pill (1-delcosine, 2-isotalatizidine, 3-talatizamine, 4-benzoylmesaconitine, 5-aconosine, 6-benzoylaconine, 7-foresticine, 8-benzoylhypaconitine, 9-songorine).

In this study, the established UPLC-ESI-MS fingerprint analysis method was used in the qualitative analysis of alkaloids in *Xiaohuoluo pill* (Figure 4A). Meanwhile, a chromatographic profiling approach using the MarkerLynx ^XS^ software was employed. There was no significant difference between the concentrated pill and the honey pill type of *Xiaohuoluo pill*. Nine alkaloids, namely, delcosine, isotalatizidine, talatizamine, aconosine, benzoylmesaconitine, benzoylaconine, foresticine, benzoyl- hypaconitine, and songorine, were picked up from the chromatogram (Figure 4B). The result indicates that the main components of the *Xiaohuoluo pill* are *Aconitum* alkaloids, including BAC, BMA, and BHA. In addition, because of their high toxicity, amounts of the three highly toxic diester-diterpene alkaloids AC, MA, and HA must be limited in the *Xiaohuoluo pill.* Therefore, the development of a rapid, valid, and sensitive method to simultaneously determine the six *Aconitum* alkaloids in the *Xiaohuoluo pill* is very important.

### 2.2. Quantitative Analysis of Xiaohuoluo Pill

#### 2.2.1. Optimization of UPLC-MS Conditions

**Figure 5:**
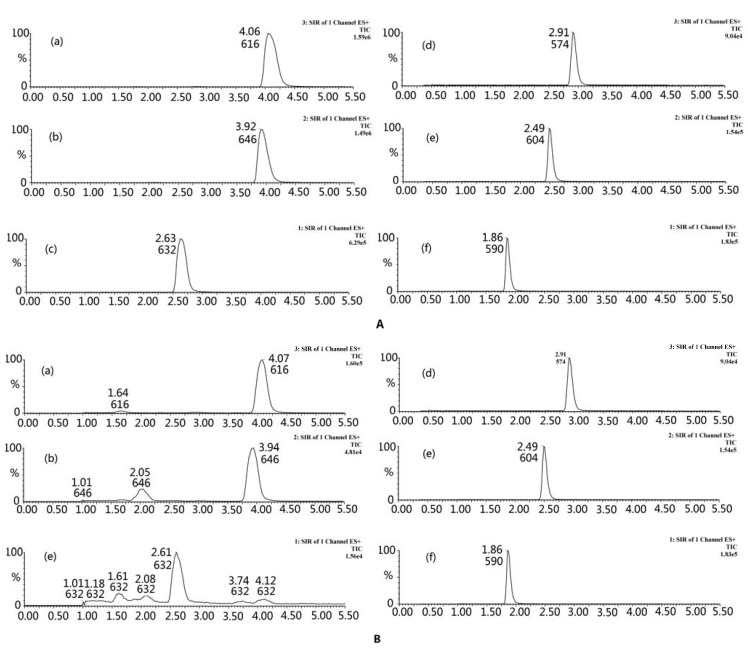
Typical SIR chromatograms of (**a**) HA, (**b**) AC, (**c**) MA, (**d**) BHA, (**e**) BAC, and (**f**) BMA. (**A**) Mixed standards; (**B**) samples of *Xiaohuoluo pill*.

In the method development, both positive and negative ion modes were investigated. Results demonstrated that the positive ion response was much higher than the negative ion one for MA, AC, HA, BMA, BAC, and BHA, which might be attributed to the ionization of the nitrogen atom of the alkaloid. Therefore, the positive ion mode was chosen in this experiment. According to the full scan mass spectra, [M+H]^+^ at 632, 646, 616, 590, 604, and 574 *m/z* were selected as precursor ions of the six analytes, respectively (Figure 5). No cross-talk interference among three channels was observed. Other parameters, such as desolvation temperature, source temperature, capillary and cone voltage, and the flow rate of desolvation gas and cone gas, were also optimized to obtain the highest intensity.

Chromatographic conditions were optimized to obtain high sensitivity, resolution, and short run time. Acetonitrile, instead of methanol, was employed in the mobile phase as it yields higher signal-to-noise (S/N) ratios. Acids are generally acknowledged to enhance ionization efficiency and improve peak shape. As such, 0.1% formic acid, rather than acetic acid, was adopted in this study. Finally, acetonitrile water containing 0.1% formic acid (35:65, v/v) was found to be the optimal mobile phase for quantitative analysis, resulting in excellent resolution, as well as peak shapes and short run time.

#### 2.2.2. Method Validation: Linearity, Limits of Detection, and Quantification

Standard stock solutions containing the six analytes were prepared and diluted to appropriate concentrations for the construction of calibration curves. The calibration curves were generated by plotting the peak areas versus the concentration of each analyte. All calibration curves showed good linearity with high correlation coefficient (*r* ≥ 0.9984) in the tested range. The lower limit of detection (LLOD) and the lower limit of quantification (LLOQ) were determined at S/N ratios of about 3 and 10, respectively. The calculated results are summarized in Table 3.

**Table 3:** Regressive equation, linear ranges, LLODs, and LLOQs of the six analytes.

Analyte	Regressive equation ^a^	*r*	Linear range (ng·mL^−1^)	LLOD ^a ^(ng·mL^−1^)	LLOQ ^b ^(ng·mL^−1^)
MA	*y* = 135.01*x* + 309.64	0.9993	1.41–501.00	0.45	1.41
AC	*y* = 54.337*x* – 21.315	0.9999	1.20–396.80	0.39	1.20
HA	*y* =148.34*x* – 130.21	0.9999	1.92–533.18	0.65	1.92
BMA	*y* = 6385.8*x* + 1390.3	0.9984	1004.00–20080.00	0.82	4.28
BAC	*y* = 15.025*x* + 314.98	0.9989	1.99–2490.00	0.54	1.99
BHA	*y* = 17.273*x* + 129.08	0.9993	2.02–1518.00	0.51	2.02

^a^ The regression equations were presented as *y* = a*x* + b*y* and *x* were defined as peak area and concentrations of analyte, respectively; ^b^ *R* = correlation coefficient, *n* = 6.

#### 2.2.3. Validation: Precision, Repeatability, Stability, and Recovery

In Table 4, precision, based on peak area measurements of six bioactive components, was better than 3.11% (RSD, *n* = 6). The reproducibility (RSD, *n* = 6) of the proposed method, based on six replicate injections, was in the range of 1.79%–3.78%. Stability (RSD, *n* = 6) of measurements over three days for the six compounds was 1.05%–3.09%. Recovery test was done using the standard addition method. Low, medium, and large amounts of the standards were added to the known sample. Extraction and analysis were then performed (Section 3.5). Mean recovery was calculated based on the following formulas: 


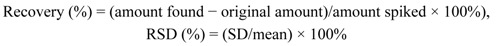


Mean recovery of the six bioactive compounds was 99.7%–101.7% and RSD value was 1.06%–2.65%. The established method was therefore sufficiently accurate in the determination of the six alkaloids in the *Xiaohuoluo pill*.

**Table 4:** Precision, reproducibility, stability, and recovery of the six analytes.

Compound	Precision RSD (%) (*n* = 6)	Reproducibility RSD (%) (*n* = 6)	Stability RSD (%) (*n* = 6)	Recovery (%) (*n* = 9)Mean ± RSD (%)
MA	2.40	2.26	1.76	99.8 ± 2.49
AC	2.36	3.78	1.42	100.9 ± 2.65
HA	2.82	1.79	1.11	101.7 ± 1.94
BMA	3.11	2.56	1.44	100.3 ± 1.06
BAC	3.07	3.07	1.05	99.7 ± 1.32
BHA	2.89	2.50	3.09	98.1 ± 1.08

#### 2.2.4. Sample Analysis

The validated assay was applied subsequently in the simultaneous determination of the six alkaloids in *Xiaohuoluo pill* purchased from different enterprises in mainland China. Each sample was analyzed in triplicate to determine the mean content (μg·g^−1^). The results are listed in Table 5. According to the package insert, the crude drug in a 1.0 g concentrated pill is equal to a 4.6 g honey pill. As such, we can deduce that the difference of the contents of the six alkaloids is in the same level.

The results in Table 5 indicate that the total of the three analytes (MA, AC, and HA) in all the samples is lower than established in the Chinese Pharmacopoeia (2010 Edition) and is safe for people if used based on the recommended usage and dosage. Moreover, the total contents of the diester-diterpenoid alkaloids (MA, AC, and HA) were obviously less than those of the monoester alkaloids (BMA, BAC, and BHA) in the *Xiaohuoluo pill*. This result indicates that traditional processing methods can improve the transformation of diester-diterpenoid alkaloids, increase the hydrolyzate content, and reduce drug toxicity.

**Table 5:** The contents (μg·g^−1^) of six targets in nine commercial samples (n = 3).

	Analytes/Contents (μg·g^−1^)					
Samples	MA	AC	HA	BMA	BAC	BHA
S-01	0.31 ± 0.01	7.55 ± 0.16	8.05 ± 0.10	295.20 ± 6.20	88.37 ± 2.16	45.45 ± 0.55
S-02	N.D. ^a^	0.18 ± 0.003	0.67 ± 0.01	327.18 ± 7.71	62.10 ± 0.53	54.75 ± 2.03
S-03	0.60 ± 0.02	0.78 ± 0.01	5.22 ± 0.12	343.38 ± 1.03	27.46 ± 0.21	52.02 ± 0.23
S-04	0.02 ± 0.002	0.07 ± 0.01	0.13 ± 0.01	28.52 ± 2.32	3.57 ± 0.29	3.11 ± 0.24
S-05	0.24 ± 0.004	2.15 ± 0.02	2.16 ± 0.02	55.71 ± 0.82	11.12 ± 0.13	9.49 ± 0.01
S-06	0.11 ± 0.003	0.13 ± 0.01	0.35 ± 0.01	14.71 ± 0.80	1.28 ± 0.05	1.34 ± 0.08
S-07	0.18 ± 0.003	0.80 ± 0.004	0.96 ± 0.01	117.80 ± 8.21	13.31 ± 1.09	11.61 ± 0.60
S-08	0.19 ± 0.002	0.84 ± 0.01	0.70 ± 0.01	137.56 ± 4.35	16.47 ± 0.40	14.34 ± 0.44
S-09	0.19 ± 0.002	0.83 ± 0.01	0.99 ± 0.01	129.54 ± 1.43	15.33 ± 0.01	13.36 ± 0.01

^a^ N.D.: higher than LLOD and less than LLOQ.

## 3. Experimental

### 3.1. Chemicals and Reagents

MA, AC, HA, BMA, BAC, and BHA were purchased from the National Institute for the Control of Pharmaceutical and Biological Products (Beijing, China) with a purity of ≥98.0%. Acetonitrile ((TEDIA, Fairfield, OH, USA)) and formic acid (Merck, Darmstadt, Germany) were of HPLC grade. Deionized water used in the experiment was prepared using the Milli-Q water purification system (Millipore, Bedford, MA, USA). Other reagents were of analytical grade and are available commercially.

### 3.2. Materials

The crude drugs and the prepared slices of *Aconitum* used in this study were collected from Sichuan province in China. These samples were authenticated and found to be the roots of *Aconitum carmichaeli Debx*. and *Aconitum kusnezoffii* *Reichb.* by Associate Professor Li-Hong Wu of the Shanghai R&D Centre for Standardization of Chinese Medicines. The finished *Aconitum* products, known as *Xiaohuoluo pill* (concentrated pill and honey pill), were purchased from different drugstores (China), and the sources are shown in Table 6.

**Table 6:** Sources of all the samples (*Xiaohuoluo Pill*) used in this work.

Name	No.	Source	Batch No.
Concentrated pill	S-01	Foci, Lanzhou	10B5
	S-02	Taibao, Lanzhou	62101204
	S-03	Dalu, Shandong	110101
Honey pill	S-04	Wohua, Shandong	110302
	S-05	Shiyitang, Harbin	1101147
	S-06	Tongrentang, Beijing	0013070
	S-07	Hongjitang, Jinan	1011001
	S-08	Hongjitang, Jinan	1104001
	S-09	Hongjitang, Jinan	1104002

### 3.3. UPLC-ESI-MS Conditions

#### 3.3.1. UPLC-MS Qualitative Conditions

Analyses were obtained based on an ACQUITY system (Waters, Milford, MA, USA), which consisted of a binary solvent manager, a cooling autosampler, and a column oven. Chromatographic separations were performed on an Agilent Waters BEH-C_18_ column (100 mm × 2.1 mm, 1.7 μm), which was maintained at 50 °C. Mobile phase consisted of acetonitrile (A) and 10 mmol ammonium-formate (0.1% ammonia, B), with the gradient elution as follows: 0–4 min, 12%–25% A; 4–7 min, 25% A; 7–12 min, 25%–40% A; 12–13 min, 40%–45% A; 13–16 min, 85% A; and 16–17 min, 95% A. Flow rate was 0.3 mL·min^−1^ and, using the partial loop mode, injection volume was 5 μL. 

Column effluents were monitored using a Micromass ZQ 2000 single quadrupole mass spectrometer (Waters, Milford, MA, USA) equipped with an electrospray ionization (ESI) source. Full-scan spectra were recorded by centroid mode from *m/z* 100–1000, at a scan time of 0.3 s and an inter-scan delay of 0.1 s. ESI-MS was performed in the positive mode under the following operating parameters: capillary voltage, 3.0 kV; cone voltage, 33.0 V; extractor voltage, 3.0 V; RF lens, 0.1 V; source temperature, 150 °C; desolvation temperature, 400 °C; desolvation gas, 650 L·h^−1^; and cone gas, 50 L·h^−1^.

#### 3.3.2. UPLC-MS Quantitative Conditions

In the above qualitative conditions, quantitative conditions were changed as follows: chromatographic separations were performed on an Agilent ZORBAX SB-C_18_ column (100 mm × 2.1 mm, 1.8 μm) and maintained at 35 °C. Mobile phase consisted of 0.1% aqueous formic acid and acetonitrile (65:35, v/v). The flow rate was 0.3 mL·min^−1^.

Waters ZQ 2000 single quadrupole mass spectrometer was also employed in selected ion recording (SIR) mode to generate *m/z* 632, 646, 616, 590, 604, and 574 for MA, AC, HA, BMA, BAC, and BHA with the same dwell time of 0.3 s. ESI-MS was performed in the positive mode under the following operating parameters: capillary voltage, 3.0 kV; cone voltage, 30.0 V; extractor voltage, 3.0 V; RF lens, 0.1 V; source temperature, 120 °C; desolvation temperature, 300 °C; desolvation gas, 500 L·h^−1^; and cone gas, 30 L·h^−1^. The MassLynx^TM^ XS software and the QuanLynx program (Waters, Manchester, UK) were used to control the UPLC-ESI-MS system, and for data acquisition and processing.

### 3.4. Preparation of Standard and Quality Control Samples

Previous studies indicated that six *Aconitum* alkaloids are not stable in a pure methanol solution and easily decompose. Therefore, standard stock solutions were prepared separately in acetonitrile, with concentrations of 0.5 mg·mL^−1^ for MA, AC, HA, BMA, BAC, and BHA, respectively. All stock solutions were stored at 4 °C, away from light, and brought to room temperature before use. Working solutions were prepared by diluting the stock solutions serially with acetonitrile until they were used.

### 3.5. Preparation of Sample Solution

#### 3.5.1. Xiaohuoluo Pill

The powder of the *Xiaohuoluo pill* was weighed accurately (approximately 0.5 g) in a stoppered conical flask, followed by the addition of ammonia TS (0.6 mL, 40% NH_3_·H_2_O), soaking it completely for 5 min. Likewise, the honey pill is cut into little pieces and weighed accurately, placing 2.3 g directly into a stopper conical flask. Then, ammonia TS (1.0 mL, 40% NH_3_·H_2_O) is added, soaking it completely for 5 min. Subsequently, each sample is extracted using a mixture of isopropanol-ethyl acetate (25 mL, 1:1, v/v) in an ultrasonic bath for 30 min [1,27]. The solution is filtered using filter paper and 1.0 mL is evaporated with nitrogen at room temperature until it is dry. The residue is reconstituted with 1.0 mL acetonitrile. The resulting solution is filtered through a 0.22 μm membrane before injecting it into the UPLC-ESI-MS system for analysis.

#### 3.5.2. *Aconitum* Herbs and Processed Products

The powders of *Aconitum* herbs and processed products are weighed accurately (approximately 1.0 g) in a stopper conical flask, followed by the addition of ammonia TS (2 mL, 40% NH_3_·H_2_O), soaking it completely. Subsequently, each sample is extracted using diethyl ether (25 mL) in an ultrasonic bath for 30 min [1,27]. The solution is filtered using filter paper and eluted with 10, 10, and 5 mL of diethyl ether. Then, the eluate is collected and evaporated on a water bath until it is dry. Using a volumetric flask, dissolve the residue in exactly 10 mL of methanol as the test solution. Finally, the resulting solution is filtered through a 0.22 μm membrane before injecting it into the UPLC-ESI-MS system for analysis.

## 4. Conclusions

Our currently developed approach provided a sensitive and applicable tool for simultaneous determination of *Aconitum* alkaloids in *Aconitum* herbs and associated preparations. The analytical method was validated and proved to be usable in terms of selectivity, linearity, accuracy and precision of MA, AC, HA, BMA, BAC, and BHA without time-consuming or expensive clean-up steps. The established method can be used as a reliable method in evaluating the quality of *Aconitum* and its contained preparations.
